# Coordinate-Based Clustering Method for Indoor Fingerprinting Localization in Dense Cluttered Environments

**DOI:** 10.3390/s16122055

**Published:** 2016-12-02

**Authors:** Wen Liu, Xiao Fu, Zhongliang Deng

**Affiliations:** School of Electronic Engineering, Beijing University of Posts and Telecommunications, No. 10 Xitucheng Road, Haidian District, Beijing 100876, China; liuwen@bupt.edu.cn (W.L.); dengzhl@bupt.edu.cn (Z.D.)

**Keywords:** indoor positioning, fingerprinting, access point deployment, clustering algorithm, K-means, coordinate based clustering, smallest enclosing circle

## Abstract

Indoor positioning technologies has boomed recently because of the growing commercial interest in indoor location-based service (ILBS). Due to the absence of satellite signal in Global Navigation Satellite System (GNSS), various technologies have been proposed for indoor applications. Among them, Wi-Fi fingerprinting has been attracting much interest from researchers because of its pervasive deployment, flexibility and robustness to dense cluttered indoor environments. One challenge, however, is the deployment of Access Points (AP), which would bring a significant influence on the system positioning accuracy. This paper concentrates on WLAN based fingerprinting indoor location by analyzing the AP deployment influence, and studying the advantages of coordinate-based clustering compared to traditional RSS-based clustering. A coordinate-based clustering method for indoor fingerprinting location, named Smallest-Enclosing-Circle-based (SEC), is then proposed aiming at reducing the positioning error lying in the AP deployment and improving robustness to dense cluttered environments. All measurements are conducted in indoor public areas, such as the National Center For the Performing Arts (as Test-bed 1) and the XiDan Joy City (Floors 1 and 2, as Test-bed 2), and results show that SEC clustering algorithm can improve system positioning accuracy by about 32.7% for Test-bed 1, 71.7% for Test-bed 2 Floor 1 and 73.7% for Test-bed 2 Floor 2 compared with traditional RSS-based clustering algorithms such as K-means.

## 1. Introduction

Indoor location-based service (ILBS) has gained considerable attention in recent years due to its social and commercial values, with market value predicted to worth US $10 billion by 2020 [1]. Meanwhile, the demand for accurate localization in indoor environments, such as large public places, office buildings with mass obstacles and military facilities, has increased dramatically [2,3]. Due to the coverage limitation of satellite signals, which are widely used in Global Navigation Satellite Systems (GNSS), various indoor positioning technologies, consisting of Infrared, Ultrasonic, Wireless Local Area Network (WLAN), Bluetooth, ZigBee, Radio Frequency Identification Devices (RFID), Pseudolite and Ultra Wideband (UWB), have been proposed to provide better performance in indoor localization. Infrared and Ultrasonic positioning technology could achieve centimeter-level accuracy in Line-Of-Sight environments [4,5], however, these two technologies both need the implementation of dense access points (AP) and the obstacles in indoor environments would have a considerable influence on the accuracy and robustness of the system. Pseudolite and UWB positioning technology can perform a centimeter-level accuracy at the cost of expensive special devices and high system complexity [6,7]. Among WLAN, Bluetooth, ZigBee and RFID positioning technologies, WLAN has aroused researchers’ interests because of its wide implementation, high mobility, low networking cost and high compatibility in dense cluttered indoor environments [8,9,10,11].

Although indoor positioning systems based on WLAN have advantages over the systems based on other technologies in dense cluttered environments, there are still some factors that can influence the systems’ performance in positioning. Positioning accuracy, robustness, computational burden, energy consumption, and the cost of the system implementation are the main factors which should to be considered when evaluating the performance of the whole system. Gu, Y. et al. [12] have given an overview and comparison on the existing WLAN-based indoor positioning systems in terms of positioning accuracy, robustness and cost. Bisio, I. et al. [13] have given a performance comparison of a probabilistic fingerprint-based indoor positioning utilizing two smartphones in terms of positioning accuracy, robustness and consistency, and has achieved relatively high accuracy about 1.2 m in a real test environment. Bisio, I. et al. [14] have proposed a novel probability computation method for indoor WiFi-based positioning to reduce the system’s computational burden, and has achieved a significant 90% decrease on energy/time consumption with mobile devices. Related works concerning the evaluation of the systems’ accuracy, computational burden and energy efficiency have also been conducted in [15,16], and these works have given important guidance for researchers on deploying fingerprint-based positioning systems. This paper mainly considered the positioning accuracy and robustness when evaluating indoor fingerprint-based positioning systems, and has given focus on the influence of AP deployment.

Indoor positioning technology based on WLAN can be divided into two different schemes: time delay-based and radio map-based. Time delay-based methods, such as Time of Arrival (TOA), Time Difference of Arrival (TDOA) and Round Trip Time (RTT), could achieve relatively higher accuracy in a relatively clean indoor environment, compared to radio map-based method. However, time delay-based methods require network synchronization and the delay should be measured exactly, which brings the localization system more complexity and is hard to realize in dense multipath and NLOS environments. Radio map-based methods, for example Received Signal Strength (RSS) based method, utilize matching algorithms to locate unknown points (UPs). Although, to a great extent, the RSS database of radio map-based methods depends on environment, these methods can achieve a relatively robust and well positioning accuracy in dense cluttered environments once the database is established.

Traditionally, RSS-based positioning methods have two stages: off-line stage and on-line stage. In the first stage, which can also be called training stage, the data collection for training is performed semi-automatically or automatically. The collected data can also be called fingerprints, which contain information representing the indoor environment. The database, also named radio map, is built based on these fingerprints. A full database should contain the coordinates (*x*, *y*, *z*) of each fingerprint, together with the Medium Access Control (MAC) addresses for each hearable AP and the corresponding RSS [17]. In the second stage, also called estimation stage, the localization of UPs is estimated by matching the RSS between UPs and fingerprints in database utilizing matching algorithms, e.g., K Nearest Neighbor (KNN). The on-line matching stage can proceed both on the server and the mobile devices, and it demands real-time signal processing. When the accuracy demand is high but the positioning area is huge, the database may be so large that the computational complexity of the matching algorithm is very high, which will bring more resource consumption and high power dissipation. Therefore, in order to reduce the computational complexity of the whole system, clustering methods are always necessary in the off-line training stage [18,19].

The deployment of APs in WLAN based positioning system has a significant influence on the accuracy of the system for most indoor environments, especially for large-scale indoor environments. In [20], an AP planning method for indoor positioning system has been proposed, together with the GDOP evaluation, in order to decrease the position error. In [21], a map-assisted AP placement algorithm has been proposed, and an accuracy within 3 m of 71% has been achieved. In [22], AP topology has been evaluated and optimized utilizing Cramér-Rao Lower Bound calculation method. The deploying schemes in [20,21,22] have an instructional significance to the implementation of APs when a new indoor WLAN based positioning system is set up. However, the APs’ deployments in most positioning conditions are generally fixed together with the construction of these infrastructures. These deployments are typically optimized for communication scope, but not for localization. This paper focused on the conditions above, analyzed the probable deployment influence on system’s accuracy and proposed a novel coordinate-based clustering method to reduce positioning error instead of changing the existed AP deployments.

We had previously proposed a smallest-enclosing-circle-based clustering algorithm for indoor positioning in [23]. This paper is an extension of the work in [23], and, in this paper, the AP implementation influence is detailed analyzed. In particular, when compared to [23], in this paper, the smallest-enclosing-circle-based clustering algorithm is described and derived in detail, and new experiments are conducted in different environments to demonstrate the effectiveness of the method proposed.

## 2. AP Deployment Influence Analysis

The deployment of APs in indoor WLAN based positioning system has a significant influence on the positioning accuracy. This section analyzes the influence brought by the implementation of APs and gives guidance about error reduction without changing existed AP deployments.

### 2.1. Indoor Wireless Signal Transmission Model

Path-loss transmission model is a theoretical mathematical model, which has been widely used to represent the transmission principle of wireless signals, such as WiFi, Bluetooth, et al., in indoor environments. As shown in Equation (1), the path-loss transmission model indicates the relationship between the transmission loss and distance of wireless signals, which assumes Line-Of-Sight (LOS) along the signal transmission route in most cases. Aiming to analyzing the influence of AP deployment in this section, this paper makes the same assumption that the transmission route between APs and Reference Points (RPs) is LOS or has little obstacles. We make the assumption for the reason that the APs in most of our experimental environments are all on the ceilings and the RPs are all on the floors. In order to make our analysis comprehensive but not complicated, we utilize the path-loss model as shown in Equation (1) with the assumption elaborated above.

(1) $$PL_{d}=PL_{0}+10n\log\frac{d}{d_{0}}+X_{\sigma },$$

where *PL_d_* is the path-loss in dBm of the wireless signals after transferred a distance of *d*. *PL*_0_ is the path-loss after a distance of *d*_0_ and *n* is an environment-depended constant which represents the loss coefficient of the indoor environment. *X_σ_* is a normally distributed variable representing the shadowing error and its distribution function is:

(2) $$X_{\sigma }∼N\left(0,\sigma^{2}\right),$$

(3) $$f\left(X_{\sigma }\right)=\frac{1}{\sqrt{2\pi }\sigma }e^{-\frac{X_{\sigma }^{2}}{2\sigma^{2}}},$$

This model shows the path-loss of wireless signals in indoor environments is of direct proportion to the logarithm of transmission distance. In general, the RSS in the fingerprint database is the received signal strength of wireless signal, which can be shown as follows:

(4) $$RSS=P+PL_{d},$$

where *P* is the transmitted power of AP. Without loss of generality, we can assume that *d*_0_ is 1 m, and it can be derived from Equations (1) and (4) that:

(5) $$RSS=A+10n\log d+X_{\sigma },$$

(6) $$A=P+PL_{0},$$

It can be concluded from Equation (5) that RSS is also of direct proportion to the logarithm of distance, like path-loss. Therefore, if the RSS of two reference points (RPs) is similar with each other, their distances to one same AP are also similar, which can be shown as follows:

(7) RSS∝logd,

(8) $$\Delta RSS=10n\left(\log d_{11}-\log d_{12}\right)=10n\log\frac{d_{11}}{d_{12}},$$

(9) $$\Delta RSS\to 0\Leftrightarrow d_{11}\approx d_{12},$$

where *d*_11_ is the distance between *AP*_1_ and *RP*_1_, and *d*_12_ is the distance between *AP*_1_ and *RP*_2_.

### 2.2. Database Structure for Indoor Positioning System

Indoor RSS based positioning system locates UPs by matching their RSS to the RSS stored in the database, and calculating their positions through matching algorithm. The database is established in the off-line training stage, when the reference points’ coordinates and RSS from APs with different MACs are measured and stored. The structure of the database generally used in positioning system can be shown as follows:

$$\left[\begin{array}{cccccc} x_{1} & y_{1} & RSS_{11} & RSS_{12} & \cdots & RSS_{1p}\\ x_{2} & y_{2} & RSS_{21} & RSS_{22} & \cdots & RSS_{2p}\\ \vdots & \vdots & \vdots & \vdots & \ddots & \vdots \\ x_{N} & y_{N} & RSS_{N1} & RSS_{N2} & \cdots & RSS_{Np} \end{array}\right]$$

where *N* is the number of RPs, and *p* is the number of APs. *RSS_ij_* is the received signal strength at *i*-th RP from *j*-th AP.

Matching algorithms calculate the location of UPs by matching their RSS to the RSSs stored in the database. Traditional matching algorithms, like KNN and Weighted-KNN (WKNN), utilize Euclidean Distance between UPs and RPs as the matching principle, which can be calculated as follows:

(10) $$dist_{Eu}\left(UP,RP_{1}\right)=\sqrt{{\left(RSS_{01}-RSS_{11}\right)}^{2}+{\left(RSS_{02}-RSS_{12}\right)}^{2}+\cdots {\left(RSS_{0p}-RSS_{1p}\right)}^{2}},$$

(11) $$dist_{Eu}\left(UP,RP_{k}\right)=\sqrt{{\left(RSS_{01}-RSS_{k1}\right)}^{2}+{\left(RSS_{02}-RSS_{k2}\right)}^{2}+\cdots {\left(RSS_{0p}-RSS_{kp}\right)}^{2}},$$

(12) $$dist_{Eu}\left(UP,RP_{k}\right)=\sqrt{\sum_{i=1}^{p}{\left(RSS_{0i}-RSS_{ki}\right)}^{2}},$$

where *dist_Eu_(UP*,*RP_k_)* is the Euclidean Distance between UP and *k*-th RP. KNN matching algorithm calculates the Euclidean distance between UP and RPs, and selects k RPs with minimum distance and chooses the barycenter as the location of UP.

### 2.3. AP Deployment Influence

In most indoor environments covered with WiFi signals, the deployment of APs (in other words, the wireless routers) is already constructed and fixed. These deployments are typically optimized for communication scope, but not for localization. Therefore, the APs’ implementation is arranged concentrated and well-organized, generally in several straight lines or Z-shaped lines above on the ceiling. This paper focuses on the situation that two RPs in the database have similar RSS and analyzed the probable reasons of this situation. This phenomenon can be divided into two different situations to analyze: the APs are close to the RPs or the APs are far from the RPs. The following subsections aim to analyze these situations.

#### 2.3.1. APs Are Close to RPs

**Figure 1:**
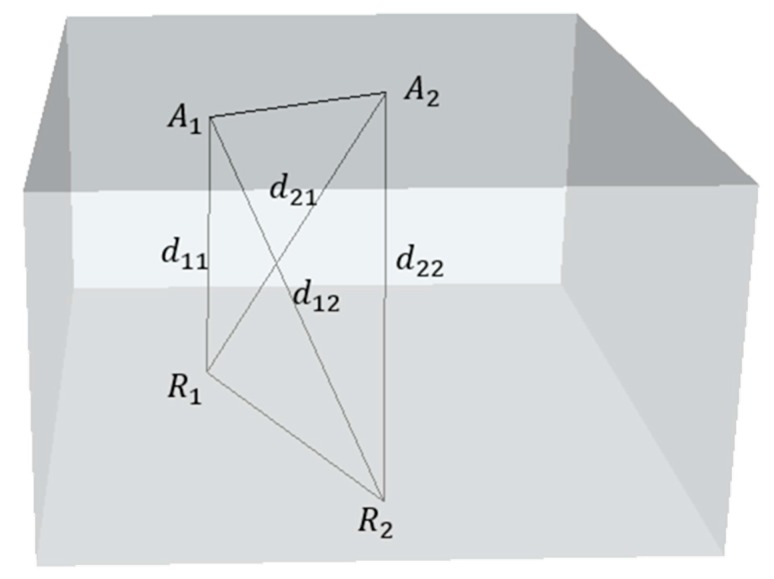
Access Point (AP) Deployment—Case 1.

When the APs above on the ceiling are close to the RPs down on the floor, there is one probable situation that can lead to the phenomenon above, which can be described as follows in Figure 1.

where *A*_1_ and *A*_2_ are the two APs above on the ceiling, and *R*_1_ and *R*_2_ are the two RPs in the database. *d*_11_, *d*_12_, *d*_21_ and *d*_22_ are the distances between APs and RPs. When the line *A*_1_*A*_2_ is orthogonal to the line *R*_1_*R*_2_ and it is also the bisector of the line *R*_1_*R*_2_ in physical space, as shown in the figure, the distance between *A*_1_ and *R*_1_ is similar with the distance between *A*_1_ and *R*_2_ according to the mid-perpendicular theorem:

(13) $$d_{11}\approx d_{12},$$

Especially, *d*_11_ is equal to *d*_12_ when the line *A*_1_*A*_2_ is exactly the mid-perpendicular of line *R*_1_*R*_2_. Thus, the RSSs of *R*_1_ and *R*_2_ in the database are similar to each other for these APs according to Equation (9).

#### 2.3.2. APs Are Distant from RPs

**Figure 2:**
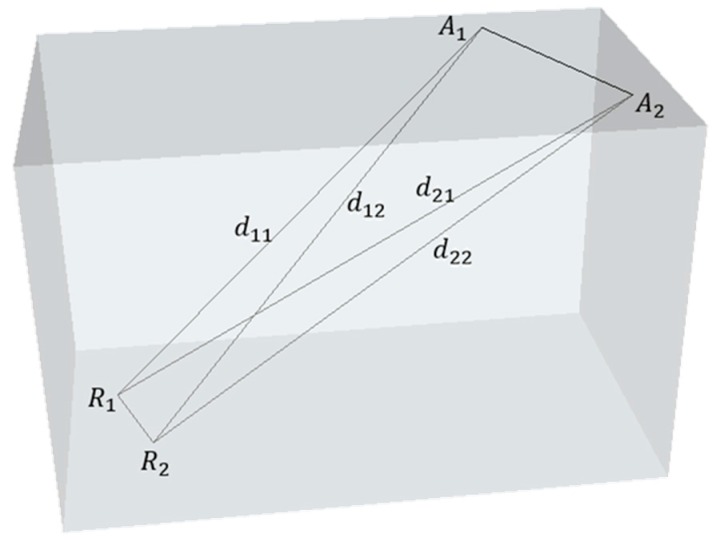
AP Deployment—Case Two.

**Figure 3:**
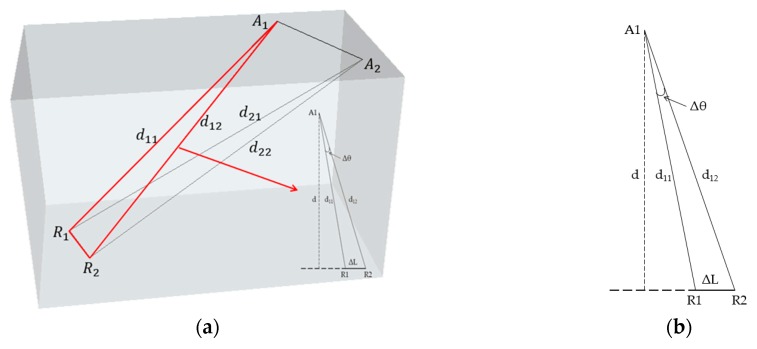
(**a**) AP deployment in Case 2 in three-dimension model; and (**b**) two-dimensional model extracted from three-dimensional model for analysis.

Instead of the APs close to the RPs, there are some APs deployed far from the RPs, which can be shown as follows in Figure 2.

where the definition of symbols are the same as the above subsection. Compared to the APs close to *R*_1_ and *R*_2_, *A*_1_ and *A*_2_ in this figure are distant from *R*_1_ and *R*_2_. The reason why the value of RSS is similar in this situation can be discussed as follows. Firstly, a two-dimensional model is extracted from the three-dimensional model, which can be shown in Figure 3.

It can be derived according to the trigonometric function theorem as follows:

(14) $$d_{11}=\frac{d}{\cos\theta_{1}}\;d_{12}=\frac{d}{\cos\left(\theta_{1}+\Delta \theta \right)},$$

(15) $$\Delta L=d_{12}\sin\left(\theta_{1}+\Delta \theta \right)-d_{11}\sin\theta_{1}=d\left(\tan\left(\theta_{1}+\Delta \theta \right)-\tan\theta_{1}\right),$$

(16) $$\Delta d=d_{12}-d_{11}=\frac{d}{\cos\left(\theta_{1}+\Delta \theta \right)}-\frac{d}{\cos\theta_{1}},$$

(17) $$\begin{array}{ll} \frac{\Delta L}{d} & =\left(\tan\left(\theta_{1}+\Delta \theta \right)-\tan\left(\theta_{1}\right)\right)\\ & =\frac{\sin\left(\theta_{1}+\Delta \theta \right)\cos\left(\theta_{1}\right)-\cos\left(\theta_{1}+\Delta \theta \right)\sin\left(\theta_{1}\right)}{\cos\left(\theta_{1}+\Delta \theta \right)\cos\left(\theta_{1}\right)}\\ & =\frac{\sin\left(\Delta \theta \right)}{\cos\left(\theta_{1}+\Delta \theta \right)\cos\left(\theta_{1}\right)} \end{array},$$

(18) $$\frac{\Delta L}{d_{12}}=\frac{\sin\left(\Delta \theta \right)}{\cos\left(\theta_{1}\right)},$$

(19) $$\frac{\Delta L}{d_{11}}=\frac{\sin\left(\Delta \theta \right)}{\cos\left(\theta_{1}+\Delta \theta \right)},$$

When the APs are distant from RPs, in other words,

(20) $$\Delta L\ll d_{11}\;and\;\Delta L\ll d_{12},$$

(21) $$\frac{\Delta L}{d_{11}}=\frac{\sin\left(\Delta \theta \right)}{\cos\left(\theta_{1}+\Delta \theta \right)}\to 0,$$

(22) $$\frac{\Delta L}{d_{12}}=\frac{\sin\left(\Delta \theta \right)}{\cos\left(\theta_{1}\right)}\to 0,$$

(23) $$\frac{\Delta L}{d_{11}}=\frac{\sin\left(\Delta \theta \right)}{\cos\left(\theta_{1}+\Delta \theta \right)}\approx \frac{\sin\left(\Delta \theta \right)}{\cos\left(\theta_{1}\right)}=\frac{\Delta L}{d_{12}},$$

then Δθ is very small and, in this situation, the distance *d*_11_ is similar with *d*_12_. Because the values of RSSs in the database are of direct proportion to the logarithm of the distance, the variation of RSS is slower than the distance. Especially when the distance between access points and reference points is very large, the variation of the RSS becomes very small. Therefore, the RSSs of RPs in the database are approximately equal according to Equation (9).

#### 2.3.3. Influence Analysis

For the reasons analyzed in previous subsections, the phenomenon that two RPs have similar RSSs in the database generally exists in indoor fingerprint positioning system. This paper named this phenomenon Singular RSS Phenomenon (SRP) to describe the existence of two RSS-similar RPs. Meanwhile, in the off-line training stage of fingerprinting positioning, clustering algorithms are conducted to achieve sparsification of database in order to reduce the system’s computation complexity of the on-line estimation stage. Traditional clustering algorithms utilized in training stage are based on the RSS in database, in other words, the algorithms cluster fingerprints into several clusters based on the comparison of their RSSs stored in database. In such conditions that SRP exists, traditional clustering algorithms would cluster these two RPs into the same cluster. In on-line estimation stage, matching algorithms are utilized to calculate the position of UPs. Matching algorithms first compare RSS between UP and cluster-heads to match the UP into one cluster, then conduct the comparison in the cluster to find the position of UP. If UP is matched into the cluster where SRP exists, the positioning results would hop between these two RPs in SRP, which brings a large bias in positioning accuracy.

## 3. Coordinate-Based Clustering

Since the deployment of APs in Wi-Fi-covered indoor environments has been fixed, and traditional clustering algorithms are not robust enough for indoor positioning, this paper proposed a novel clustering method based on the coordinates of RPs together with their RSS. Coordinate-based clustering methods can be divided into two schemes generally: the grid-based clustering and grid-free clustering.

### 3.1. Grid-Based Clustering

Grid-based clustering method has been proposed in [24], and has achieved a relatively high accuracy and robustness in indoor fingerprinting positioning. The clustering algorithms based on meshing first divide experimental environments into several grids with the same size. Then, the RPs in database are clustered into the closest grid based on the distance principle. These grid-based clustering is one of the coordinate-based clustering, and can be more robust than RSS-based clustering in environments where SRP exists. However, one challenge of the clustering algorithms based on meshing is that the size of grid should be specified before clustering and is always fixed. Therefore, grid-based clustering may not be flexible in different indoor dense cluttered environments.

### 3.2. Smallest-Enclosing-Circle-Based Clustering

Smallest enclosing circle (SEC) algorithm has been widely investigated in information security field [25], such as data searching and information filtering, whereas it has not been introduced into indoor fingerprint positioning system for clustering to date. This subsection analyzes the method of SEC and proposes the coordinates-based clustering algorithm, which introduces the SEC algorithm into database clustering.

#### 3.2.1. Smallest Enclosing Circle Model

Smallest enclosing circle of a points set can be described as follows:

(24) $$B\left(c,r\right):=\left\{x:\left\|x-c\right\|\leq r;x\in \Omega ;r=r_{\min}\right\},$$

where *B*(*c*, *r*) is the circle with minimum radius that consists of all the points in the given set, and *x* and *c* represents the point *x* and the center of circle *B*, respectively. In general, the center of circle is assumed *O_m_* with coordinates (*x_m_*, *y_m_*) and radius *R_m_*. The lines connect the center of circle and the points *k*_1_, *k*_2_, *k*_3_, …, *k_n_* inside of the circle intersect the circle with point *P_i_* (*i* = 1, 2, …, *n*). The SEC problem is to estimate the (*x_m_*, *y_m_*) and *R_m_* that minimize the objective function, given by Equation (10).

(25) $$f\left(d\right)=\sum_{i=1}^{n}\left|O_{m}P_{i}-O_{m}k_{i}\right|,$$

where *O_m_* is the center of circle, and the definitions of *P_i_* and *k_i_* have been given above.

#### 3.2.2. Smallest Enclosing Circle Clustering Algorithm

SEC clustering algorithm, as one of the grid-free clustering algorithms, is proposed to compensate the error introduced by conventional clustering algorithms such as K-means. This algorithm clusters the reference points in fingerprint database into several clusters (this paper takes *k* to represent the number of clusters) based on their coordinates in physical space, instead of their RSS values. This can mitigate the positioning area deviations in dense cluttered indoor environments.

The number of clusters *k* in SEC clustering algorithm is selected equal to or less than the square root of RPs’ number, which is the same selection principle as K-means clustering algorithm in [26]. In general, a well clustering method should follow the principle that the distances among cluster-heads are larger than the distances between RPs and their cluster-heads in its clustering results. This paper takes the mean distance of inter-cluster and intra-cluster to elaborate the selection principle of number of clusters in SEC clustering algorithm. This paper takes the symbol *L* and *D* to represent the inter-cluster and intra-cluster distance, respectively, which can be shown as follows:

(26) $$L=\sum_{i=1}^{k}\sum_{j=1}^{k}\left|O_{i}O_{j}\right|\;\left(i\neq j\right),$$

(27) $$D=\sum_{i=1}^{k}\sum_{P\in C_{i}}\left|PO_{i}\right|,$$

where *k* is the number of clusters, *O_i_* represents the cluster-head in the *i*-th cluster *C_i_* and *P* represents the RPs in cluster *C_i_*. Assuming that there are *n* RPs in fingerprint database, then the mean inter-cluster and intra-cluster distance can be defined as follows:

(28) $$\bar{l}=L/k,$$

(29) $$\bar{d}=D/n,$$

In order to obtain relatively well clustering results, the intra-structure of one cluster is similar with the inter-structure of clusters, and the inter-cluster distance is larger than the intra-cluster distance [26], which can be shown as follows:

(30) $$\frac{\bar{d}}{D/k}\leq \frac{\bar{l}}{L},$$

It can be derived from Equations (28)–(30) that the selection principle of *k* in SEC clustering algorithm can be shown as Equation (31).

(31) $$k\leq \left\lfloor \sqrt{n}\right\rfloor ,$$

The experimental evaluation of the influence of different numbers of clusters on the positioning system will be further discussed in Section 4, and without loss of generality, this paper selects the number of clusters *k* equal to the square root of RPs’ number *n*. The implement of the SEC algorithm can be given as follows.

**A. Symbol Definition:**

The definition of symbols used in SEC clustering algorithm are as follows in Table 1.

(32) $$k=\left\lfloor \sqrt{Card\left(P\right)}\right\rfloor$$

**B. Algorithm Process:**

The proposed clustering algorithm based on smallest enclosing circle consists of four steps, which can be processed as follows:

Step 1. Select *k* points from set *P* randomly for the initialization of smallest enclosing circle-based algorithm.
Step 2. Calculate the Euclidean distances between remaining points and the selected points in database. Cluster the remaining points into *k* smallest enclosing circles based on the minimum-distance principle, and update the centers and radiuses of circles based on the theory of SEC.
Step 3. Evaluate the changes of circle centers before and after SEC algorithm, if the center changes are larger than the given threshold, then do Step 2; else do Step 4.
Step 4. Store the centers’ coordinates and RSS information into database for on-line evaluation stage.

The pseudo code of the clustering process is as follows in Algorithm 1.
**Algorithm 1:** Smallest Enclosing Circle (SEC) Clustering Algorithm**Initialize:**$\forall P_{k}=\left\{p_{1},p_{2},\cdots ,p_{k}\right\}\subseteq P$for(i=1,i≤k,i++)$\{O_{i}=p_{i};r_{i}\leq \min\left(dist\left(p_{a},p_{b}\right)\right);$$\left(p_{a},p_{b}\in P;a\neq b\right);C_{i}=\left(O_{i},r_{i}\right);\}$**Calculation:**
for(i=1,i≤k,i++){for(j=k+1,j≤n,j++)$\{D_{ij}=dist\left(p_{i},p_{j}\right);D_{im}=\underset{j}{\min}\left(D_{ij}\right);$$p_{m}\in C_{i};\}$$if\left(dist\left(p_{m},O_{i}\right)\leq r_{i}\right)$$\left\{r_{i}^{\prime }=r_{i};O_{i}^{\prime }=O_{i};C_{i}^{\prime }=C_{i}≜\left\{\left(O_{i},r_{i}\right)\right\};\right\}$$else\{O_{i}^{\prime }=mid\left(O_{i},p_{m}\right);$$r_{i}^{\prime }=r_{i}+\frac{1}{2}dist\left(O_{i},p_{m}\right);C_{i}^{\prime }=\left\{\left(O_{i}^{\prime },r_{i}^{\prime }\right)\right\};\}$**Verification:**
$if\left(dist\left(O_{i}^{\prime },O_{i}\right)\leq \delta \right)break;$
$else\{O=O^{\prime };R=R^{\prime };C=C^{\prime };$
$repeat\;step\;2\;until\left(dist\left(O_{i}^{\prime },O_{i}\right)\leq \delta \right);\}$

## 4. Measurement Analysis

### 4.1. Experiments Scenario and Implementation

We conducted our experiments in two dense cluttered environments, one is the public area at the National Center For the Performing Arts (denoted as Test-bed 1), and the other is at XiDan Joy City, which is a multi-story shopping mall in Beijing (denoted as Test-bed 2).

**Figure 4:**
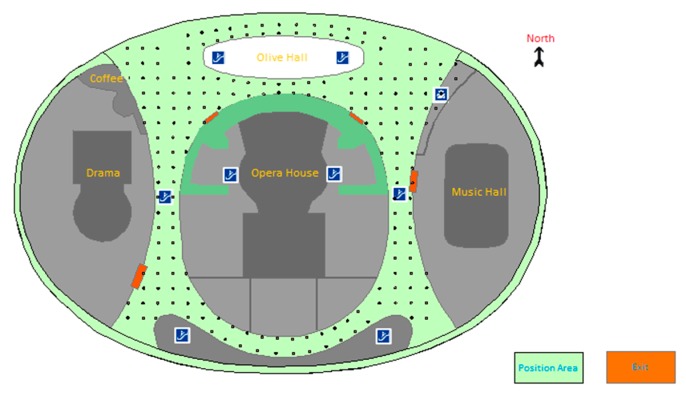
Experimental environments for Test-bed 1.

Figure 4 shows the planar graph and the deployment of RPs in Test-bed 1 with a plotting scale of 1:2800. The area marked as green, with an area of 210 m by 140 m, is the public area where our experiment is conducted. This area is covered with IEEE 802.11b/g wireless signals from 58 wireless routers with unique MAC addresses. The deployment of these wireless routers (called Access Points) is ordered as Z-shaped lines. The points marked as black in Figure 4 represent the reference points stored in the fingerprint database, with an interval of 5 m. The number of points for training stage is 260, and the RSS information stored at one point is an average value of 100 times measurements. Hence, 26,000 pieces of RSS data are collected for calculation of average value, and 260 pieces of RSS data are stored in database finally.

**Figure 5:**
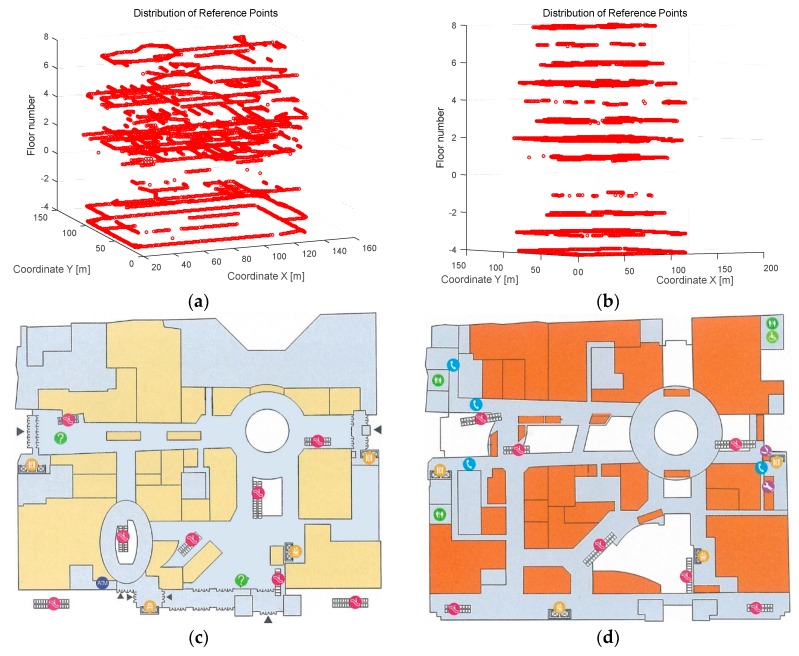
Experimental environments for Test-bed 2: (**a**) reference points’ distribution in Test-bed 2, twelve floors in total; (**b**) reference points’ distribution in Test-bed 2 in side-view; (**c**) the first floor of Test-bed 2 in planar graph; and (**d**) the second floor of Test-bed 2 in planar graph.

Figure 5 shows the reference points’ implementation of Test-bed 2 at XiDan Joy City. XiDan Joy City is a multi-story shopping mall with 12 floors (eight floors above-ground and four floors under-ground) at XiDan, Beijing. The experimental environment for Test-bed 2 is with an area of 120 m by 90 m, which is covered with IEEE 802.11b/g wireless signals. There are 4949 reference points in this experiment for all the 12 floors and 10 pieces of RSS data are collected at each point utilizing a cellphone, which runs our collecting program at RPs to collect the RSSI from different APs. The RSS data stored in the database for each point is the average of these 10 pieces of data, which are collected from four different orientations. The distribution of the total 4949 RPs at 12 floors and the number of hearable APs at each floor is shown in Table 2. We have conducted our experiments at each floor in XiDan Joy City. However, in order to validate the effectiveness of SEC algorithm analyzed above well, we selected floor-one and floor-two as the test-bed to discuss our clustering results, in consideration of the number of RPs or APs in each floor and the AP deployment in each floor. The clustering results and positioning error comparisons in other floors are also shown in this paper at the Appendix. Figure 5 shows the experimental scenarios of floor-one and floor-two, in which floors the APs were deployed along as several straight lines.

### 4.2. Measurement Results and Analysis

#### 4.2.1. Clustering Results and Analysis

**Figure 6:**
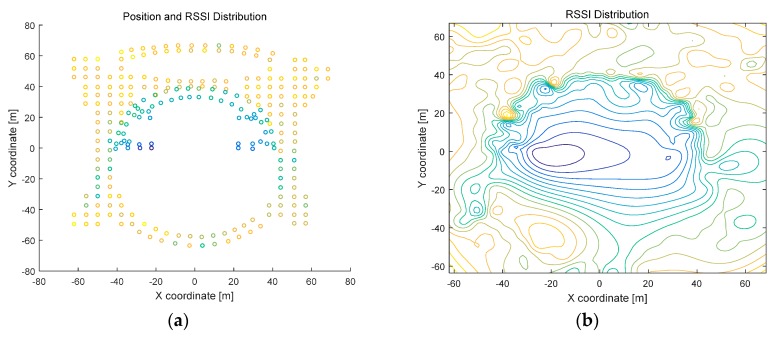
Reference points’ Received Signal Strength Indicator (RSSI) distribution in Test-bed 1: (**a**) RSSI distribution in scatter diagram; and (**b**) RSSI distribution in contour map.

**Figure 7:**
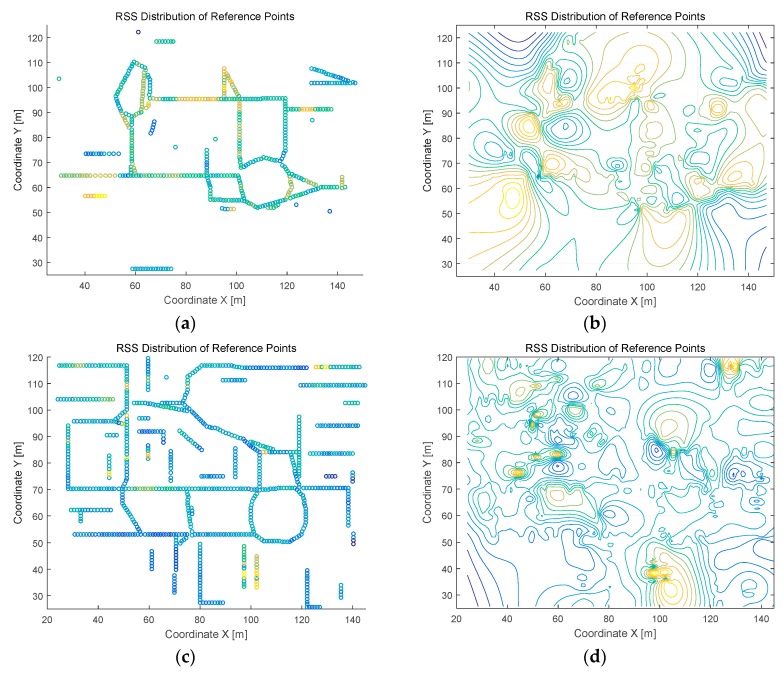
Reference points’ RSSI distribution in Test-bed 2: (**a**) RSSI distribution in the first floor of Test-bed 2 in scatter diagram; (**b**) RSSI distribution in the first floor of Test-bed 2 in contour map; (**c**) RSSI distribution in the second floor of Test-bed 2 in scatter diagram; and (**d**) RSSI distribution in the second floor of Test-bed 2 in contour map.

The distributions of the RPs and their RSS values in experimental fingerprint database are investigated, and the distribution results both in scatter diagram and contour diagram are presented in Figure 6 (for Test-bed 1) and Figure 7 (for Test-bed 2). The contour diagram is based on the method of linear interpolation. It can be concluded from Figure 6 and Figure 7 that the distribution of wireless signals in dense cluttered environments is very complex and full of randomness. In the contour diagrams, the points on the same contour line have similar RSS value in the database. Therefore, some distant points in physical space are close to each other in signal space, which is analyzed as SRP in Section 2.

**Figure 8:**
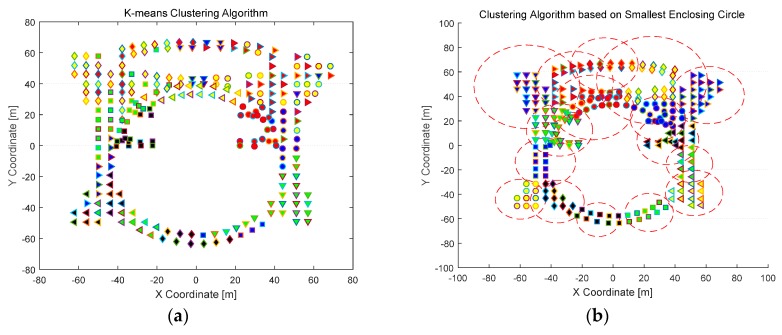
Clustering results comparison in Test-bed 1: (**a**) clustering results under K-means clustering algorithm; and (**b**) clustering results under Smallest Enclosing Circle (SEC)-based clustering algorithm.

**Figure 9:**
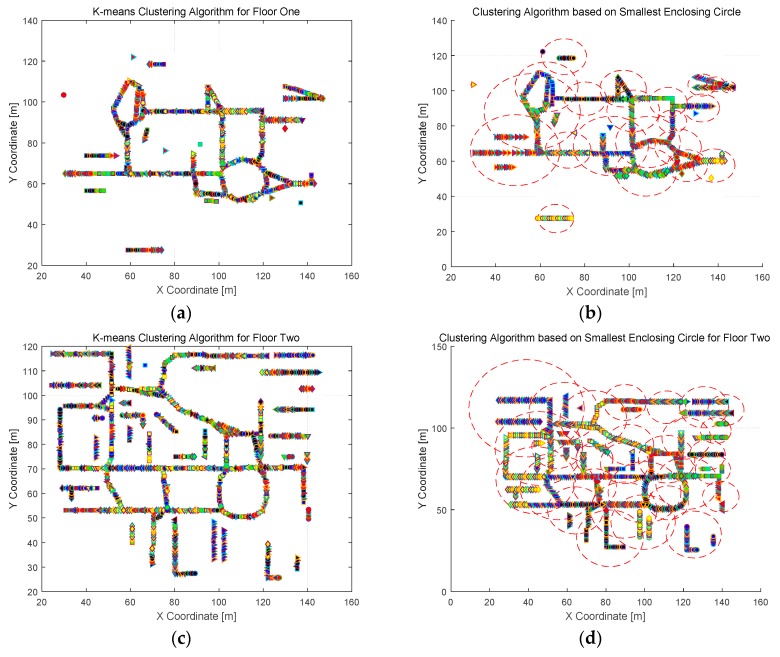
Clustering results comparison in Test-bed 2: (**a**) clustering results under K-means clustering algorithm in the first floor; (**b**) clustering results under SEC-based clustering algorithm in the first floor; (**c**) clustering results under K-means clustering algorithm in the second floor; and (**d**) clustering results under SEC-based clustering algorithm in the second floor.

Clustering comparison is conducted utilizing the methos of K-means and SEC. K-means is a classical and RSS-based clustering algorithm, and SEC is a novel and coordinate-based clustering algorithm proposed in this paper. Clustering results are shown in Figure 8 and Figure 9 for Test-beds 1 and 2, respectively. It can be concluded that the clustering results using K-means is dispersive in physical space, and SRP has a significant influence on RSS-based clustering method. On the other hand, the clustering results utilizing SEC is compact, and SEC clustering is robust to SRP. Meanwhile, the capacity of each cluster in SEC clustering results is different, which means the flexibility of SEC is better than other coordinate-based clustering methods, like grid-based clustering.

**Figure 10:**
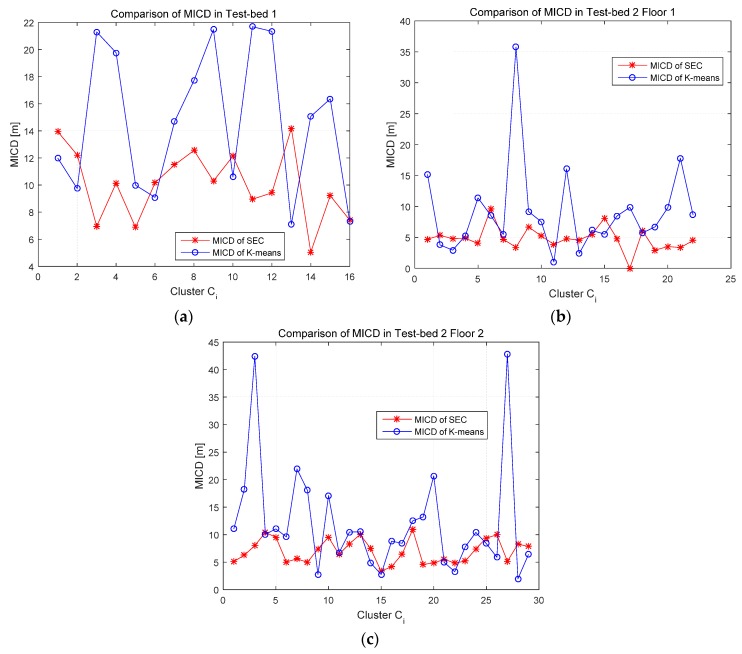
Comparison of Mean Intra-Cluster Distance (MICD) between K-means and SEC clustering: (**a**) MICD comparison in Test-bed 1; (**b**) MICD comparison in Test-bed 2 Floor 1; and (**c**) MICD comparison in Test-bed 2 Floor 2.

In order to compare the clustering results between K-means and SEC algorithm more comprehensive and to validate the effectiveness of coordinate-based clustering method, a specific metric, named Mean Intra-Cluster Distance (MICD), has been given to quantify the level of dispersion of RPs in these two clustering methods. MICD represents the mean intra-cluster distance of all the RPs in one same cluster. The calculation of MICD is shown as Equation (33) and the comparisons of MICD between K-means and SEC are shown in Figure 10.

(33) $$MICD_{k}=\left(\sum_{P\in C_{k}}\sqrt{{\left(x_{P}-x_{O_{k}}\right)}^{2}+{\left(y_{P}-y_{O_{k}}\right)}^{2}}\right)/n_{k},$$

where *MICD_k_* is the MICD of *k*-th cluster, (*x_P_*, *y_P_*) is the coordinates of the reference point *P*, and (*x_Ok_*, *y_Ok_*) is the coordinates of the *k*-th cluster-head *O_k_*. *C_k_* represents the *k*-th cluster. According to Equation (33), we calculated the MICD of clustering results using K-means and SEC. Figure 10 shows the comparisons of MICD in Test-beds 1 and 2. It can be concluded that the SEC clustering algorithm can cluster RPs more compact in physical space and more robust to SRP.

#### 4.2.2. Positioning Accuracy Results and Analysis

**Figure 11:**
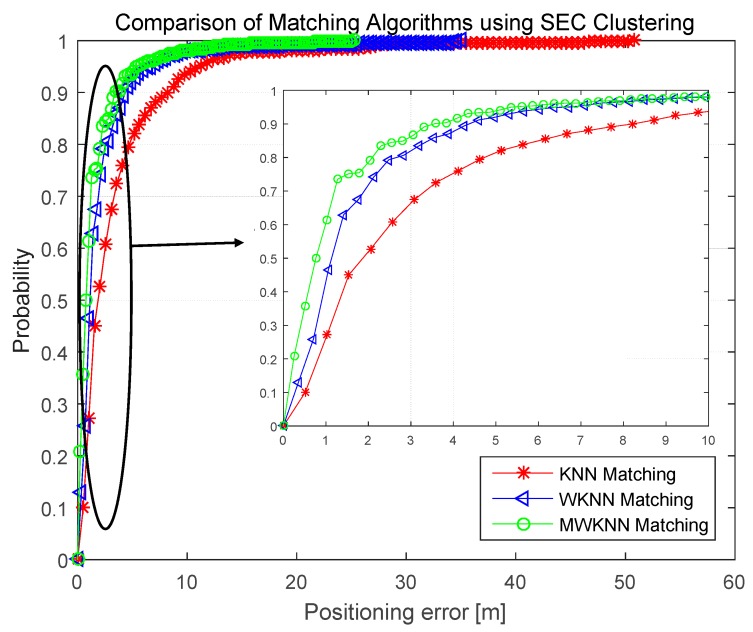
Comparison of matching algorithms using SEC clustering.

Positioning accuracy is an important evaluation metric for an indoor positioning system. The accuracy of fingerprint positioning system utilizing K-means and SEC clustering algorithms in the training stage is measured and compared for both two Test-beds. In this paper, we measured 250 test points for Test-bed 1, 500 test points for Test-bed 2 Floor 1 and 850 test points for Test-bed 2 Floor 2. All of these test points were selected randomly in the experimental scenarios, and the systems’ positioning errors were measured for analysis. Regarding to the matching algorithm of fingerprint positioning system, this paper firstly conducted three matching algorithms, namely K Nearest Neighbor (KNN), Weight K Nearest Neighbor (WKNN) and Modified Weight K Nearest Neighbor (MWKNN) [23], in Test-bed 2 Floor 1 to evaluate the performance of positioning system. The comparison of positioning errors in cumulative distribution function (CDF) for these three matching algorithms has been shown in Figure 11.

It can be concluded from Figure 11 that WKNN and MWKNN matching algorithms perform better positioning accuracy than KNN matching algorithm. Among all the 500 test points in Test-bed 1 Floor 1, there are 309 points (about 61.8%) with the positioning error within 1.02 m using MWKNN, 1.41 m using WKNN and 2.56 m using KNN. Meanwhile, there are 449 points (about 89.8%) with the positioning error within 3.31 m using MWKNN, 4.24 m using WKNN and 8.21 m using KNN. Without loss of generality, we select one same matching algorithm, namely MWKNN, for the purpose to compare the clustering performance utilizing both K-means and SEC. In the following discussion of this paper, the comparisons of positioning accuracy of these two clustering algorithms are all under the same MWKNN matching algorithm.

It should be pointed out that in Figure 11 there are still few location errors at test points (about 2% using MWKNN, 3% using WKNN and 6% using KNN) that are more than 10 m, and even some location errors (about 0.6% using MWKNN, 0.9% using WKNN, 2% using KNN) more than 20 m. There are two main reasons for these high location errors, one is the large difference of experimental environments between off-line training stage and on-line positioning stage at these test points, and the other is the large fluctuation in the transmitted power of some APs. Researchers who would implement this fingerprint positioning system could utilize other sensors’ information, such as inertial sensors, to assist and calibrate the positioning results to achieve higher positioning accuracy.

**Figure 12:**
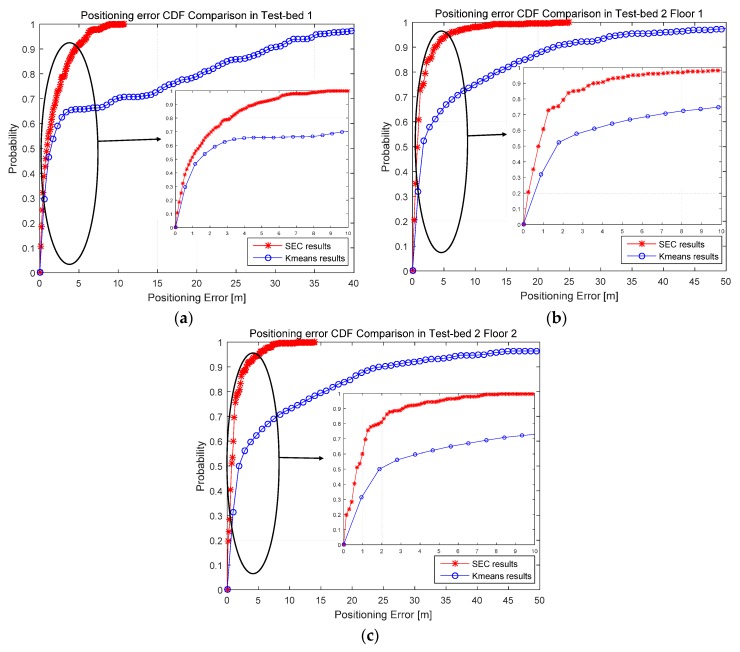
Positioning performance comparison between K-means and SEC in Cumulative Distribution Function (CDF) of positioning error under MWKNN matching algorithm: (**a**) CDF comparison in Test-bed 1; (**b**) CDF comparison in Test-bed 2 Floor 1; and (**c**) CDF comparison in Test-bed 2 Floor 2.

The comparisons of positioning performance between K-means and SEC clustering algorithm utilizing MWKNN matching have been shown in Figure 12 and Table 3. It can be concluded that the positioning accuracy has been improved by about 32.7% in Test-bed 1, 71.7% in Test-bed 2 Floor 1 and 73.7% in Test-bed 2 Floor 2, using SEC compared to K-means. It can also be concluded that coordinate-based clustering algorithm, like SEC proposed in this paper, is more robust in indoor environments when SRP exists, compared with K-means.

#### 4.2.3. Number of Clusters Evaluation

**Figure 13:**
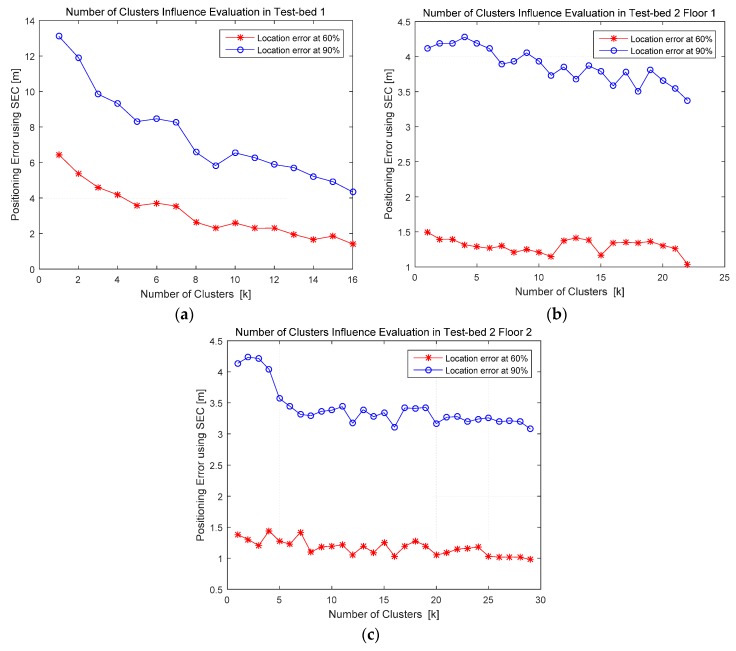
Number of clusters influence evaluation and comparison in Test-bed 1 and Test-bed 2 utilizing SEC clustering under MWKNN matching algorithm: (**a**) number of clusters influence evaluation in Test-bed 1; (**b**) number of clusters influence evaluation in Test-bed 2 Floor 1; and (**c**) number of clusters influence evaluation in Test-bed 2 Floor 2.

According to the analysis on the influence of number of clusters in Section 3, this paper conducted experiments using SEC clustering algorithm in Test-bed 1 and Test-bed 2 with different numbers of clusters. We selected different numbers of clusters, namely *k* in this paper, within its range given in Section 3, from one to the maximum. The performance of positioning systems with different *k* is measured utilizing the location error at 60% and 90% test points, and the comparisons are shown in Figure 13. It can be concluded that the selection of number of clusters has an influence on location error of the fingerprint positioning system, and within the selection range given in Section 3, the best positioning performance lies in the selection of maximum *k*, which equals to the square root of total number of RPs. Researchers who would implement these positioning systems could select number of clusters as the maximum *k* analyzed in Section 3.

### 4.3. Related Works

Many indoor fingerprint-based positioning systems over the years have been proposed to improve the systems’ positioning accuracy and robustness, which have been reviewed in [5]. RADAR system has been proposed to locating and tracking users in indoor environments in [27], and has achieved a positioning accuracy of 2.37–2.65 m at 50% and 5.93–5.97 m at 90%. Horus system, which has been proposed in [28], has offered a joint clustering technique and probabilistic method for location estimation, and has achieved an accuracy of 0.86 m and 1.32 m for their test bed 1 and test bed 2, respectively. In [21], an AP placement algorithm based on map information has been proposed to improve the system’s positioning performance in term of accuracy, and an accuracy within 3 m for 71% has been achieved. Our work has given focus on the influence of AP deployment for indoor fingerprint-based positioning system, and has proposed a coordinate-based clustering method to reduce these influences on positioning accuracy. Some of the performance comparisons of related works are shown in Table 4 and our work has been conducted also aiming at giving guidance for researchers who would implement a fingerprint-based positioning system in indoor dense cluttered environment.

## 5. Conclusions

In this paper, a novel coordinate-based clustering method, named Smallest Enclosing Circle (SEC) based clustering, is proposed, analyzed and estimated. The influence on fingerprint database and positioning accuracy of AP deployments is studied, and a probable phenomenon named SRP in dense cluttered environments is analyzed. Traditional clustering, like K-means, used in fingerprint positioning cannot satisfy the accuracy demand in these environments because of the existence of SRP. The SEC clustering is proposed to reduce the influence of AP deployment and SRP, and to improve the accuracy and robustness of the whole system in dense cluttered indoor environments. The experiments in two different real public indoor area are conducted to validate the advantages of the proposed method. All results are based on real field measurements at China National Grand Theatre and XiDan Joy City. The results show that SEC clustering is more robust and flexible compared to RSS-based clustering, and the improvement of positioning accuracy using SEC clustering is about 32.7% for Test-bed 1, 71.7% for Test-bed 2 Floor 1 and 73.7% for Test-bed 2 Floor 2, compared to RSS-based clustering. Meanwhile, the accuracy influence of number of clusters has been analyzed and evaluated by environments, and results have shown that SEC clustering performed more robust than K-means clustering in indoor environments with SRP. Our study is useful in fingerprint positioning systems in dense cluttered environments where the AP deployment is ordered and fixed, and it is also a guid for AP deployment in a new environment to implement fingerprint positioning.

## Figures and Tables

**Table 1:** Symbol definition in Smallest Enclosing Circle (SEC) clustering algorithm.

Symbol	Definition	Mathematical Operators	Definition
$P=\left\{P_{1},P_{2},\cdots ,P_{n}\right\}$	RPs in the database, *p_k_* is the *k*-th RP	⌊A⌋	Integer notation, represents the maximum integer less than *A*.
$O=\left\{O_{1},O_{2},\cdots ,O_{k}\right\}$	Circle centers in SEC, *O_k_* is the center of *k*-th circle	∀	Arbitrary notation, represents the selection is random.
*k* ^1^	Number of clusters, selected as a constant generally	*min*	Minimum selection notation.
$R=\left\{r_{1},r_{2},\cdots ,r_{k}\right\}$	Radiuses of SECs, *r_k_* is the radius of *k*-th circle	*mid(A, B)*	Midpoint calculation, represents the midpoint of the line *AB*.
$C=\left\{C_{1},C_{2},\cdots ,C_{k}\right\}$	SECs, *C_k_* is the *k*-th circle	*dist*	Distance calculation, represents the Euclidean distance in this paper.

^1^ The selection of clusters’ number *k* depends on the number of RPs in the database. In this paper, *k* is selected as a constant and can be shown as Equation (32), where *Card* function is to calculate the number of elements in set *P*, in others words, *k* equals to the positive square root of RP numbers.

**Table 2:** Distribution of RPs and the number of APs at 12 floors in Test-bed 2.

Floor Number	Number of RPs	Number of Hearable APs ^1^
Floor 1	504	110
Floor 2	893	133
Floor 3	574	86
Floor 4	140	23
Floor 5	720	115
Floor 6	385	52
Floor 7	131	27
Floor 8	349	37
Floor b1 ^2^	54	21
Floor b2	372	57
Floor b3	427	76
Floor b4	400	63

^1^ The hearable APs represent the APs with different MACs, which can be measured RSSI at one floor; ^2^ Floor b1 means the first floor under ground in Test-bed 2.

**Table 3:** Positioning Performance Comparison between K-means and SEC under Modified Weight K Nearest Neighbor (MWKNN) matching.

Positioning Error	Test-Bed 1	Test-Bed 2 Floor 1	Test-Bed 2 Floor 2
K-means	2.26 m (60%)	3.58 (60%)	3.73 m (60%)
	28.35 m (90%)	21.50 m (90%)	22.43 m (90%)
SEC	1.52 m (60%)	1.07 m (60%)	0.98 m (60%)
	4.67 m (90%)	3.78 m (90%)	3.24 m (90%)

**Table 4:** Comparison of related works.

Related Works	Positioning Accuracy	Experimental Environments
RADAR [27]	2.37 m (50%) and 5.93 m (90%)	Real testing environments
Horus [28]	0.86 m and 1.32 m	Test bed 1 (68.2 × 25.9 m^2^); Test bed 2 (11.8 × 35.9 m^2^)
Works in [21]	3 m (71%)	1200 m^2^ testing environments
Our work	1.52 m (60%) and 3.24 m (90%)	Test-bed 1 (210 × 140 m^2^); Test-bed 2 (120 × 90 m^2^)

**Figure A1:**
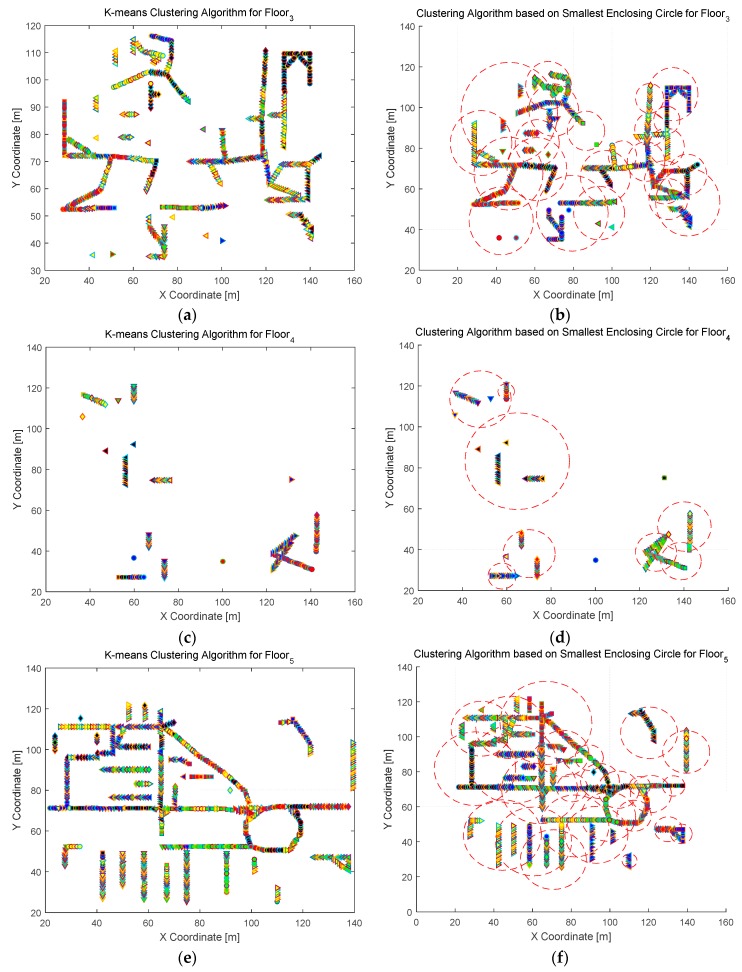

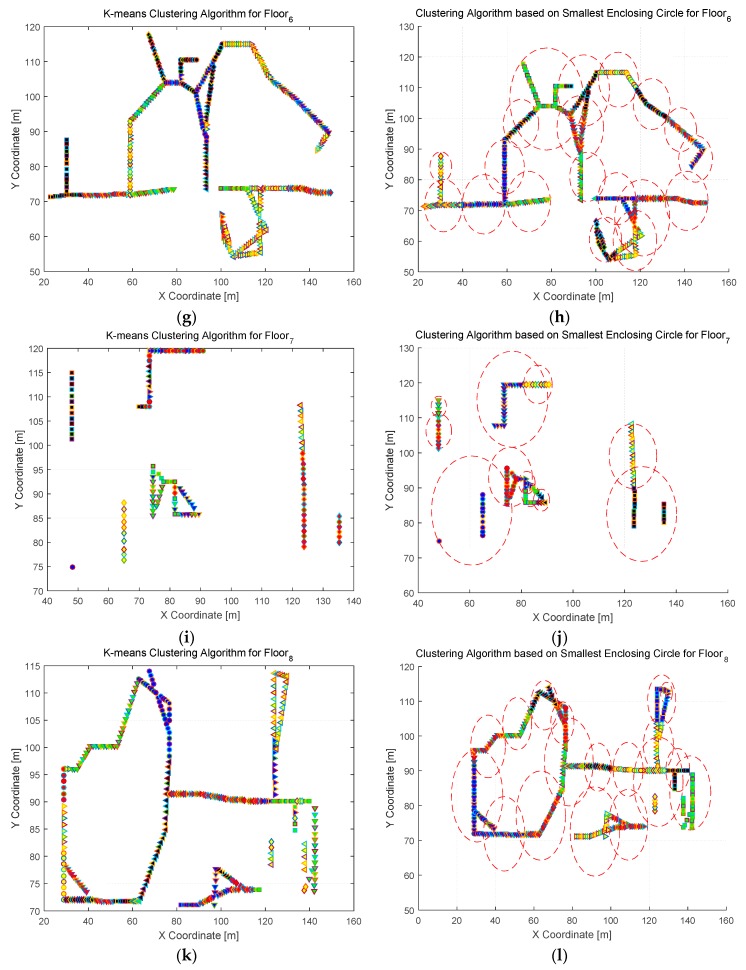

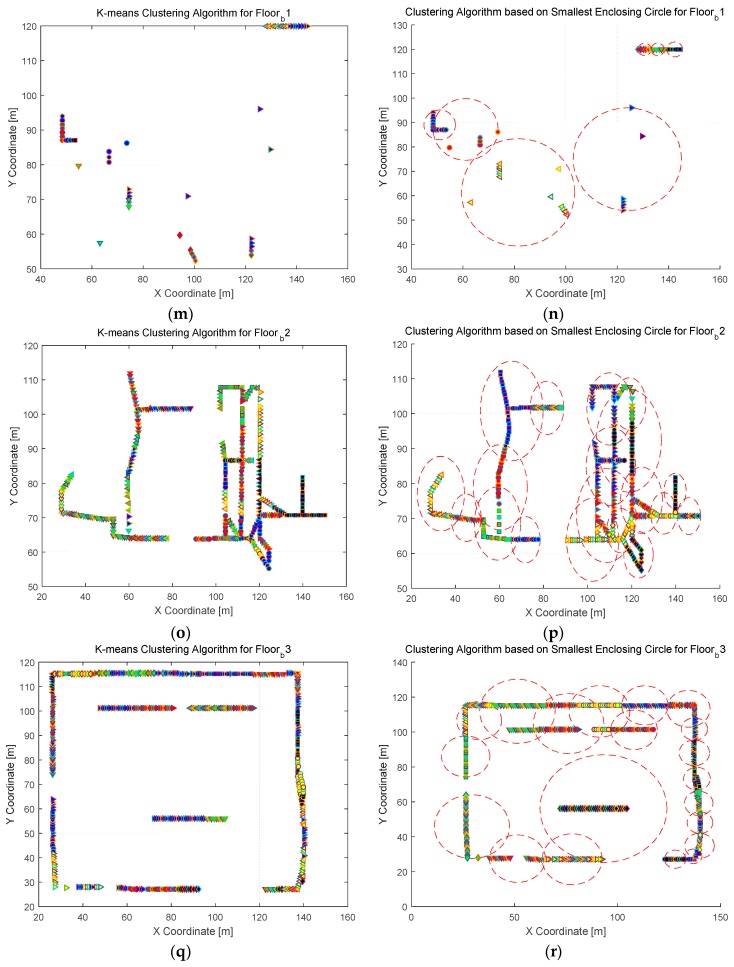

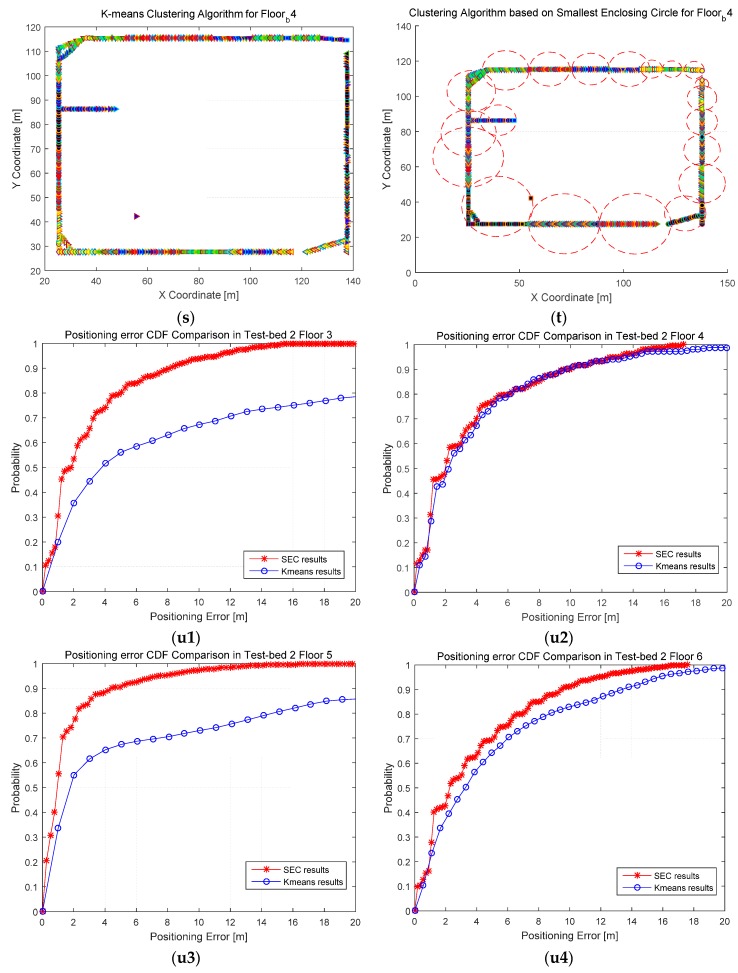

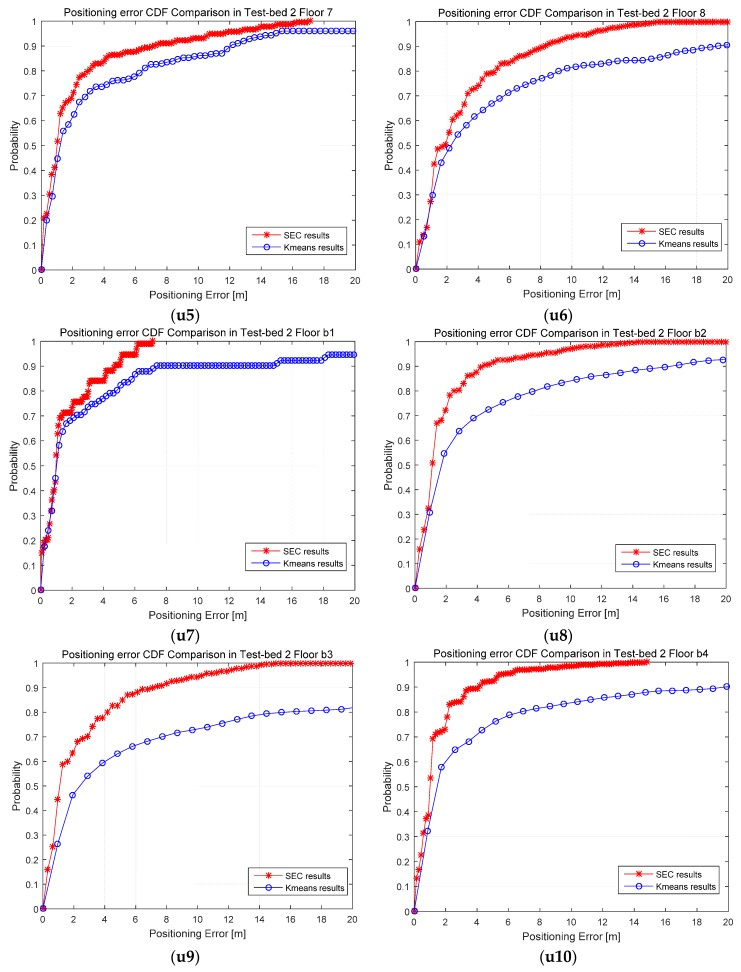
Clustering results and positioning performance comparisons for Test-bed 2: (**a**–**t**) the clustering results and comparisons between K-means and SEC from Floor 3 to Floor b4 in Test-bed 2; and (**u1**–**u10**) the positioning performance comparisons between K-means and SEC from Floor 3 to Floor b4 in Test-bed 2.

## Appendix A

This appendix is for the additional experimental results in Test-bed 2 at XiDan Joy City. The clustering results and positioning performance comparisons for the other floors are shown in in Figure A1.
